# Leucine-rich repeat kinase 2 positively regulates inflammation and down-regulates NF-κB p50 signaling in cultured microglia cells

**DOI:** 10.1186/s12974-015-0449-7

**Published:** 2015-12-09

**Authors:** Isabella Russo, Giulia Berti, Nicoletta Plotegher, Greta Bernardo, Roberta Filograna, Luigi Bubacco, Elisa Greggio

**Affiliations:** Department of Biology, University of Padova, via Ugo Bassi 58/B, 35131 Padova, Italy; Current address: Department of Cell and Developmental Biology, University College London, London, UK; Current address: Department of Laboratory Medicine, Karolinska Institute, Stockholm, Sweden

**Keywords:** LRRK2, Microglia, Neuroinflammation, Parkinson’s disease

## Abstract

**Background:**

Over-activated microglia and chronic neuroinflammation contribute to dopaminergic neuron degeneration and progression of Parkinson’s disease (PD). Leucine-rich repeat kinase 2 (LRRK2), a kinase mutated in autosomal dominantly inherited and sporadic PD cases, is highly expressed in immune cells, in which it regulates inflammation through a yet unclear mechanism.

**Methods:**

Here, using pharmacological inhibition and cultured *Lrrk2*^*−*/*−*^ primary microglia cells, we validated LRRK2 as a positive modulator of inflammation and we investigated its specific function in microglia cells.

**Results:**

Inhibition or genetic deletion of LRRK2 causes reduction of interleukin-1β and cyclooxygenase-2 expression upon lipopolysaccharide-mediated inflammation. LRRK2 also takes part of the signaling trigged by α-synuclein fibrils, which culminates in induction of inflammatory mediators. At the molecular level, loss of LRRK2 or inhibition of its kinase activity results in increased phosphorylation of nuclear factor kappa-B (NF-κB) inhibitory subunit p50 at S337, a protein kinase A (PKA)-specific phosphorylation site, with consequent accumulation of p50 in the nucleus.

**Conclusions:**

Taken together, these findings point to a role of LRRK2 in microglia activation and sustainment of neuroinflammation and in controlling of NF-κB p50 inhibitory signaling. Understanding the molecular pathways coordinated by LRRK2 in activated microglia cells after pathological stimuli such us fibrillar α-synuclein holds the potential to provide novel targets for PD therapeutics.

## Background

Mutations in the leucine-rich repeat kinase 2 (*Lrrk2*) gene cause late-onset, autosomal dominant Parkinson’s disease (PD) with clinical and pathological phenotypes almost indistinguishable from those of idiopathic disease [1, 2]. *Lrrk2* encodes a large multidomain protein belonging to the ROCO (Ras Of COmplex) family of proteins, which is characterized by the presence of a catalytic domain comprising a ROC (Ras Of Complex proteins)/GTPase, a COR (C-terminus of ROC) and a serine threonine kinase domain, and a number of repeat sequences important for protein-protein or protein-membrane interactions at both the N- and C-terminals [1, 3]. Among all identified *Lrrk2* pathological mutations, G2019S, located in the kinase domain, is by far the most frequent in both familial and apparently sporadic PD cases [4]. The G2019S mutation has attracted much attention as it robustly enhances LRRK2 kinase activity in vitro [5, 6] and in vivo [7], and this activity has been reported to be toxic to neuronal cells [5, 8]. Of interest, Sheng and colleagues observed that additional pathological mutations, other than G2019S, display increased kinase activity by monitoring LRRK2 autophosphorylation at S1292 [7], supporting the notion that the pathogenic effects of LRRK2 might be mediated by an augmented kinase activity.

LRRK2 has been linked to several pathways in neuronal cells, including vesicular trafficking [9, 10], cytoskeletal dynamics [11–13], mitochondrial functions [14, 15], apoptosis [16], and autophagy process [17, 18]. However, how LRRK2 pathogenic mutants contribute to neurodegeneration in PD remains elusive. Multiple studies reported that LRRK2 is more expressed in immune cells, especially in B cells, monocytes, macrophages, and microglia compared to T cells [19]. Furthermore, it has been found that cultured microglia display ~three- to fourfold more LRRK2 basal expression than neuronal cells [20], implying a crucial role of LRRK2 in these cells. Thus, one hypothesis is that pathological LRRK2 activity in microglia cells may impact neuronal functions as secondary event. In support of a crucial role of this protein in the immune system, genome-wide association studies identified *Lrrk2* as one of the susceptibility genes for leprosy and Crohn’s disease [21, 22], two illnesses with a significant inflammatory component. Coherently, analysis of inflammed colonic tissue from Crohn’s disease patients revealed increased levels of LRRK2 expression [19]. At the molecular level, LRRK2 has been shown to negatively control the nuclear transcription factor NFAT in bone marrow-derived macrophages and the inflammatory response [23]. Instead, in cultured microglia cells, the kinase was suggested to regulate the activity of the transcription factor nuclear factor kappa-B (NF-κB) through a yet unknown mechanism [24]. NF-κB transcription factor signaling is one of the main regulators of cyclooxygenase-2 (COX-2), interleukin-1β (IL-1β), and other pro-inflammatory mediators during inflammation [25]. The most abundant form of NF-κB is the heterodimer composed by p65 and p50 subunit [26]. Specifically, p50 is generated from the proteolytic processing of the precursor p105, it lacks the transcription activation domain, and it forms homodimers with no ability to activate gene expression [27]. In unstimulated cells, p50 is detected in the nucleus where it is primarily present as homodimer able to bind DNA and repress NF-κB-dependent gene expression [27–29]. In the canonical pathway, NF-κB p65 bound to IκBs inhibitory proteins is phosphorylated at S536 by the IKK complex upon an inflammatory stimulus. This results in IκBs proteasomal degradation and release of NF-κB p65:p50 dimers that enter the nucleus and activate transcription of target genes [30]. Thus, the activated p65:p50 heterodimers are able to bind DNA and induce gene expression by displacing the p50:p50 homodimers [27].

In this study, using the GSK2578215A (GSK) inhibitor and cultured *Lrrk2*^*−/−*^ primary microglia cells, we validated LRRK2 as a positive modulator of inflammation in microglia cells. We showed that LRRK2 takes part of the signaling trigged by α-synuclein (α-syn) fibrils, which culminates in microglia activation and induction of inflammatory IL-1β cytokine. We further demonstrated that *Lrrk2*^*−/−*^ primary microglia, under unstimulated conditions, display increased levels of nuclear and S337 phosphorylated NF-κB p50 compared to *Lrrk2*^*+/+*^ cells. We validated the increased level of p50 phosphorylation also in BV2 cells upon LRRK2 pharmacological inhibition and *ex vivo* using *Lrrk2* knock-out mouse brain lysates. Overall, our data suggest that LRRK2 kinase activity may control microglial inflammation by regulating protein kinase A (PKA)-mediated NF-κB p50 phosphorylation, which is crucial for binding and repression of NF-κB target genes. Given that chronic neuroinflammation is recognized to contribute to PD pathogenesis, understanding the specific function(s) of LRRK2 activity in microglia cells and during inflammation may disclose novel pathways for therapeutic intervention.

## Methods

### Cell cultures

BV2 cells were cultured in RPMI-40 medium (Sigma-Aldrich) supplemented with 10 % fetal bovine serum (FBS) (Life technologies), 2 mM glutamine (Sigma-Aldrich), and penicillin and streptomycin (Life technologies) and maintained at 37 °C in a 5 % CO_2_ controlled atmosphere.

### Primary microglia cell cultures

All animal procedures were performed following the guidelines issued by the European Community Council Directive 2010/63/UE and approved by the Ethics Committee of the University of Padova (Project ID: 46/2012). Microglia cells were derived from postnatal days 1–4 (P1-P4) *Lrrk2*^*+*/*+*^ and *Lrrk2*^*−*/*−*^ mouse brains (C57BL/6J). Cerebral cortices were mechanically dissociated in cold HBSS (Sigma-Aldrich), then cellular suspension was allowed to settle for 5 min, and the top fraction was collected, centrifuged for 5 min at 1000 *g*, and re-suspended in DMEM-F12, supplemented with 10 % FBS, 2 mM glutamine, 2 mM sodium pyruvate (Sigma-Aldrich), penicillin, and streptomycin. Cell suspension obtained from the three brains was plated on poly-l-lysine (0.1 mg/ml, Sigma-Aldrich) coated T-75 flask. After 4 days, the medium was replaced, and the mixed glial culture was maintained until day 14. At 14 days, microglia cells were isolated from the mixed culture by shaking 4 h at 160 rpm, and the purity of the obtained culture was verified by double immunofluorescence with mouse anti-CD11b (Cell signaling) for microglia cells and with rabbit anti-GFAP (DAKO) for astrocytes. The primary microglia yield was ~5 × 10^5^ cells/flask, and the amount of astrocyte contaminants was negligible.

### Production and aggregation of recombinant α-syn

Human α-syn fibrils were generated from recombinant α-syn produced by a lipid A mutant of *Escherichia coli*, BL21(DE3) with strongly reduced endotoxicity [31]. After purification, α-syn was incubated for 15 days to induce aggregation and quantified as previously reported [32].

### Compounds and treatments

During treatments, BV2 and primary microglia cells were cultured in medium containing 1 % FBS. Inflammation was induced using 50 EU/ml lipopolysaccharide (LPS) from *E. coli* O111:B4 (Sigma-Aldrich, L4391, potency 500,000 EU/mg) suspended in phosphate-buffered saline (PBS) or α-syn fibrils at 25 μM (monomer concentration before fibrillation). PBS (Life technologies) or α-syn monomer at 25 nM were used as control. To evaluate the effect of LRRK2 on pro-inflammatory proteins (BV2 and primary microglia cells) and on mRNAs (BV2 cells), LPS treatment was maintained for 5 h, whereas to evaluate the effect of LRRK2 on mRNAs and LPS molecular signaling (primary microglia and BV2 cells), LPS treatment was maintained for 90 min. LRRK2 inhibitors GSK and IN-1 (Tocris Bioscience) were used at 2 and 1 μM, respectively, and maintained in the medium for all the time of inflammatory treatment. Forskolin (Sigma-Aldrich) and PKI (Tocris Bioscience) were used at 30 and 20 μM, respectively, for 90 min.

### Cells and brain lysis and western blotting

Cells washed with PBS and mouse brains after dissection were solubilized with lysis buffer (20 mM Tris-HCl pH 7.5, 150 mM NaCl, 1 mM EDTA, 2.5 mM sodium pyrophosphate, 1 mM β-glycerophosphate, 1 mM Na_3_VO_4_) supplemented with 1 % Triton X-100 (Sigma-Aldrich) and protease inhibitor cocktail (Sigma-Aldrich) and then cleared at 14,000 *g* at 4 °C for 30 min. Protein concentrations were determined using the BCA protein concentration assay as manufacturer’s instructions (Thermo Scientific). Subsequently, proteins were separated by electrophoresis onto 4–20 % SDS-PAGE gels and then transferred onto Immobilon-P membrane. Membranes were incubated 1 h at room temperature (RT) with the following antibodies: rabbit anti-LRRK2 MJFF2 (1:1000, Abcam), rabbit anti-IL1β (1:1000, Santa Cruz), rabbit anti-COX-2 (1:2000, Cayman Chemical), mouse anti-GAPDH (1:2000, Millipore), mouse anti-β-tubulin (1:3000, Sigma-Aldrich), mouse α-actin (1:3000, Sigma-Aldrich), rabbit anti-p65 total (1:2000, Cell signaling), rabbit anti-phospho serine 536 p65 (1:1000, Cell signaling), rabbit anti-p105/p50 (1:2000, Cell signaling), and rabbit anti-phospho serine 337 p50 (1:1000, Santa Cruz). Subsequently, membranes were incubated 1 h at RT with HRP-conjugated secondary antibodies (Sigma-Aldrich) and finally incubated with ECL western blot substrate (Thermo Scientific).

### RNA extraction, retro-transcription and semi-quantitative PCR

BV2 and primary microglia cells were collected with TRIzol (Life Technologies) and incubated 15 min at RT. After adding chloroform, cells were incubated for 15 min and then centrifuged at 12,000 *g* for 15 min at 4 °C. Subsequently, to precipitate RNA, 100 % isopropanol was added to the aqueous phase, incubated 10 min at RT, and centrifuged at 12,000 *g* for 15 min at 4 °C. The RNA pellet was washed with 75 % ethanol, air-dried for 30 min, and then resuspended in RNAse-free water. Retro-transcription was carried out using ImProm-II Reverse Transcriptase as manufacturer’s instructions (Promega). Briefly, 1 μg RNA was mixed with 1 μl of 500 ng/μl random primers (Promega) and incubated 70 °C for 5 min and then 4 °C for 5 min. Then, reverse transcription mix was prepared with 5*X* reaction buffer (Promega), 1 μl of 100 μM dNTPs, 1 μl of 40 U/μl RNasin (Promega), and 1 μl ImProm-II RT (160 u/μl) in a final volume of 20 μl and incubated at 25 °C for 5 min and 42 °C for 1 h, and finally, the enzyme was inactivated at 70 °C for 15 min.

To analyze RNA expression, semi-quantitative PCRs were carried out using the following primer sequences (IL-1β FOR 5′-GGCAACTGTTCCTGAACTCAACTG-3′ and REV 5′-CCATTGAGGTGGAGAGCTTTCA-3′; COX-2 FOR 5′-TCAAAAGAAGTGCTGGAAAAGGT-3′ and REV 5′-GATCATCTCTACCTGAGTGTCT-3′; GAPDH FOR 5′-GAGAGTGTTTCCTCGTCCCG-3′ and REV 5′-ACTGTGCCGTTGAATTTGCC-3′). For PCR reaction, we used 20 ng of cDNA and 25 amplification cycles (linear phase of the PCR reaction) for each gene analyzed.

### Immunofluorescence and confocal imaging

Cells were washed once with PBS and fixed using 4 % paraformaldehyde for 20 min. Then, cells were permeabilized with 0.3 % Triton X-100 in PBS for 5 min and saturated with blocking solution containing 5 % FBS and 0.3 % Triton X-100 in PBS for 30 min at RT. Primary antibodies CD11b (1:100), GFAP (1:100), and p105/p50 (1:100) diluted in blocking solution were incubated 1 h at RT. After several washes, the cells were incubated 1 h at RT with secondary antibodies Alexa-fluor 488 and Alexa-fluor 546 (1:200, Life Technologies), and after repeated washes, the cells were mounted using Mowiol reagent containing Hoechst (Roche). Images were acquired with a Leica TCS SP5 confocal microscope using Zeiss 63X objective. Quantifications of nuclear p50 were performed using ImageJ software. In detail, the mean of fluorescence intensity was calculated as p50 nuclear fluorescence intensity divided by the nuclear area and expressed as nuclear intensity/μm^2^. Quantification was performed in blind, and at least ninety cells were randomly chosen in four independent experiments per genotype.

### TEM

α-Syn fibrils resuspended in PBS were absorbed onto a carbon-coated copper grid and were then negative stained with 0.05 % uranyl acetate solution. Transmission electron microscopy (TEM) micrographs were taken with a FEI Tecnai G2 12 electron microscope operating at 100 kV.

### Statistical analysis

All quantitative data are expressed as mean ± SEM and represent at least three independent sets of experiments. Statistical significance of differences between two groups was assessed by unpaired *t* test, while for multiple comparisons by one-way ANOVA with Tukey’s post- hoc test. Cumulative frequency distributions were compared with a Kolmogorov-Smirnov test. Data were analyzed using Prism (GraphPad).

## Results

### LRRK2 pharmacological inhibition attenuates pro-inflammatory mediators at protein and mRNA levels after LPS-mediated inflammation

Pharmacological inhibition of LRRK2 attenuates inflammatory response in microglia cells after LPS or HIV-1 Tat protein pathological stimuli, indicating that LRRK2 may control inflammation through its kinase activity [33, 34]. However, LRRK2 inhibitors have been shown to have off-target effects [35, 36], highlighting the need of using multiple pharmacological tools in conjunction with genetic approaches to study LRRK2 functions. To this aim, we validated the role of LRRK2 kinase activity after LPS-mediated inflammation using LRRK2-IN-1 [37] and GSK2578215A (hereafter GSK), a selective LRRK2 inhibitor [38].

**Fig. 1:**
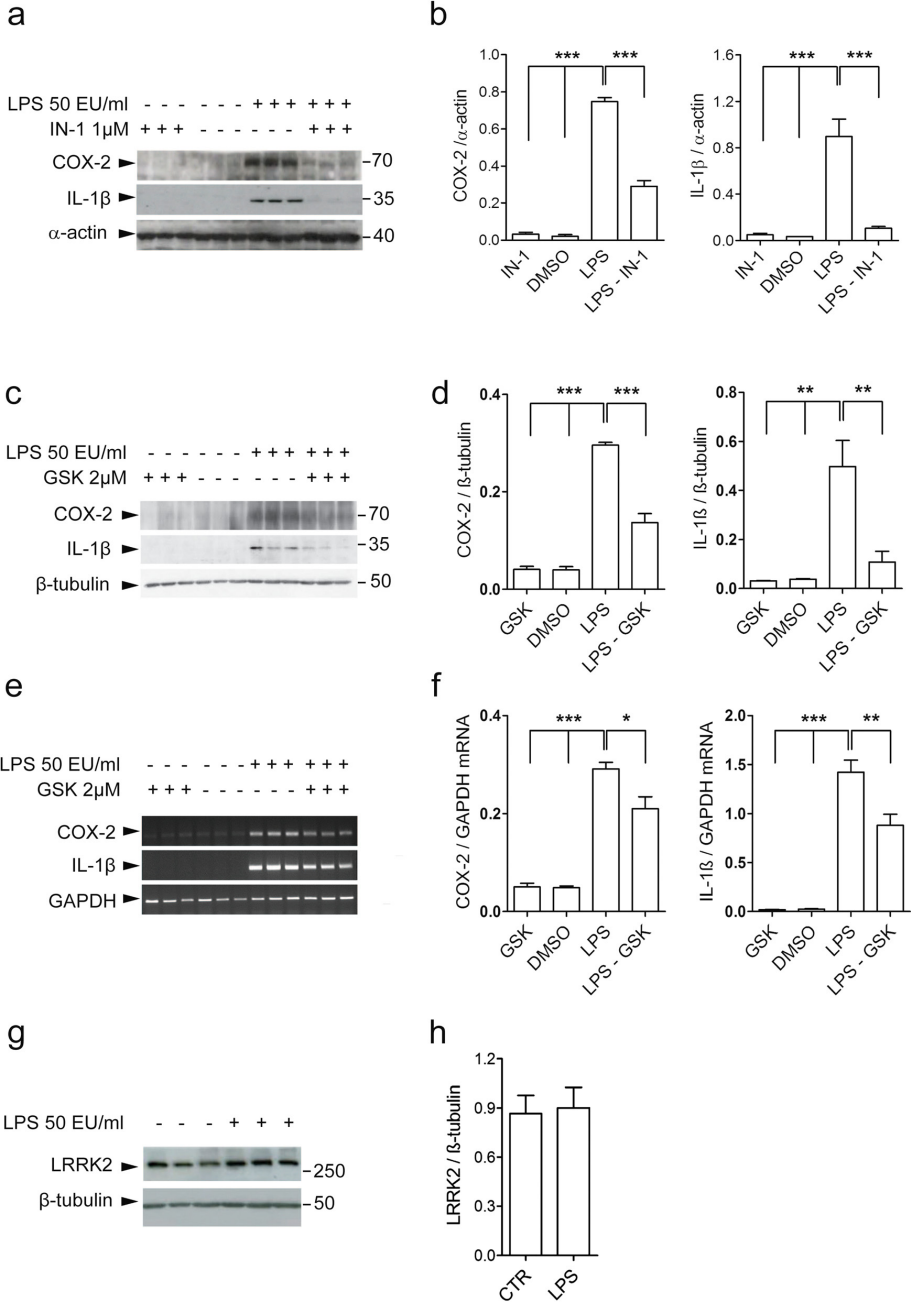
LRRK2 kinase inhibition attenuates inflammation in BV2 cells. **a** BV2 cell lysates treated with LPS, LPS and IN-1, and IN-1 alone or DMSO as control were subjected to immunoblotting using COX-2 and IL-1β antibodies. **b** Quantification of COX-2 and IL-1β are normalized for α-actin protein. Data are representative of three independent experiments (*bars* represent the mean ± SEM; one-way ANOVA comparing all groups, Tukey’s multiple comparison post hoc test; ***p < 0.001). **c** BV2 cell lysates treated with LPS, LPS and GSK, and GSK alone or DMSO as control were subjected to immunoblotting using COX-2 and IL-1β antibodies. **d** Quantification of COX-2 and IL-1β are normalized for β-tubulin. Data are representative of three independent experiments (*bars* represent the mean ± SEM; one-way ANOVA comparing all groups, Tukey’s multiple comparison post hoc test; **p < 0.01 and ***p < 0.001). **e** BV2 cell lysates treated with LPS, LPS and GSK, and GSK alone or DMSO as control were subjected to semi-quantitative PCRs. **f** Quantification of COX-2 and IL-1β mRNAs are normalized for GAPDH. Data are representative of three independent experiments (*bars* represent the mean ± SEM; one-way ANOVA comparing all groups, Tukey’s multiple comparison post hoc test; *p < 0.05, **p < 0.01 and ***p < 0.001). **g** BV2 cell lysates treated with LPS and DMSO as control were subjected to immunoblotting using LRRK2 and β-tubulin antibodies. **h** Quantification of LRRK2 protein is normalized for β-tubulin. Data are representative of six independent experiments (*bars* represent the mean ± SEM; unpaired *t* test)

To assess whether LRRK2 kinase activity controls the induction of pro-inflammatory mediators, we treated BV2 cells with LPS for 5 h in the presence of LRRK2 inhibition. We extended the incubation time of LRRK2 inhibitor to that of LPS since pro-inflammatory mediators can be generated throughout the LPS treatment time. In agreement with previous reports [33, 34], IN-1 decreases the inflammatory response as revealed by reduction of COX-2 and IL-1β precursor (hereafter IL-1β) after LPS priming (Fig. 1a, b). Importantly, we confirmed these findings with GSK inhibitor. As shown in the Fig. 1, LPS-mediated increment of COX-2 and IL-1β cytokine is attenuated in the presence of GSK, at translation (Fig. 1c, d) and transcription levels (Fig. 1e, f). Of note, LRRK2 steady state levels were unchanged upon LPS treatment (Fig. 1g, h). Taken together, these results indicate that LRRK2 kinase activity modulates the inflammatory response in microglia cells.

### *Lrrk2*^*−*/*−*^ primary microglia cells exhibit attenuated pro-inflammatory mediators after LPS priming

**Fig. 2:**
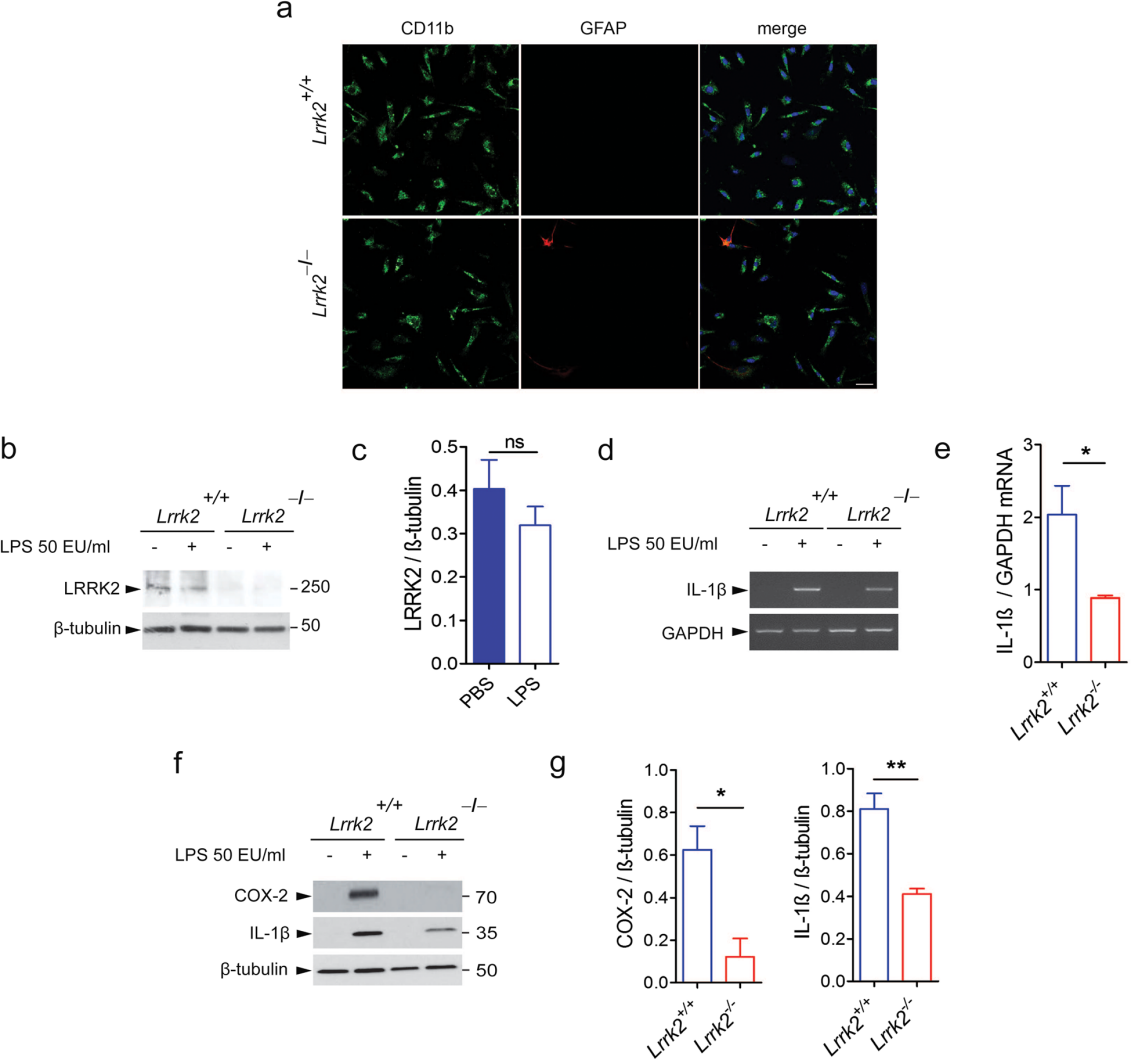
*Lrrk2* ^*−*/*−*^ primary microglia cells exhibit attenuated inflammation. **a** *Lrrk2* ^*+*/*+*^ and *Lrrk2* ^*−*/*−*^ primary microglia culture purity. Representative images of *Lrrk2* ^*+*/*+*^ and *Lrrk2* ^*−*/*−*^ primary microglia culture stained for microglial CD11b (*green*), astrocytic GFAP (*red*), and nuclei with Hoechst (*blue*). Scale bar 25 μm. **b** *Lrrk2* ^+/+^ and *Lrrk2* ^*−*/*−*^ microglia lysates treated with LPS or PBS as control were subjected to immunoblotting using LRRK2 and β-tubulin antibodies. **c** Quantification of LRRK2 protein is normalized for β-tubulin. Data are representative of three independent experiments (*bars* represent the mean ± SEM; unpaired *t* test). **d** *Lrrk2* ^+/+^ and *Lrrk2* ^*−*/*−*^ microglia lysates treated with LPS or PBS as control were subjected to semi-quantitative PCR. **e** Quantification of LPS-mediated IL-1β mRNA is normalized for GAPDH. Data are representative of three independent experiments (*bars* represent the mean ± SEM; unpaired *t* test; *p < 0.05). **f** *Lrrk2* ^+/+^ and *Lrrk2* ^*−*/*−*^ microglia lysates treated with LPS or PBS as control were subjected to immunoblotting using COX-2 and IL-1β antibodies. **g** Quantification of LPS-mediated COX-2 and IL-1β are normalized for β-tubulin. Data are representative of three independent experiments (*bars* represent the mean ± SEM; unpaired *t* test; *p < 0.05, **p < 0.01)

To validate the contribution of LRRK2 in microglia during inflammation, we investigated the response of cultured *Lrrk2*^*+*/*+*^ and *Lrrk2*^*−*/*−*^ primary microglia cells to LPS priming. Primary microglia cells were isolated from *Lrrk2*^*+*/*+*^ and *Lrrk2*^*−*/*−*^ mice. The amount of astrocyte contaminants was negligible, as showed by immunocytochemistry for CD11b-positive microglia cells and GFAP-positive cells (Fig. 2a). *Lrrk2* genetic deletion was confirmed by immunoblotting in *Lrrk2*^*+*/*+*^ and *Lrrk2*^*−*/*−*^ microglia cells (Fig. 2b). Similar to BV2 cells, primary microglia cells treated with LPS for 5 h did not exhibit a significant change in LRRK2 expression (Fig. 2b, c). However, when we analyzed the levels of LPS-mediated pro-inflammatory mediators in *Lrrk2*^*+*/*+*^ and *Lrrk2*^*−*/*−*^ microglia cells, we found that *Lrrk2*^*−*/*−*^ microglia cells displayed ~55 % reduction of IL-1β mRNA expression (Fig. 2d, e) and a reduction of ~80 % of COX-2 and 50 % of IL-1β protein levels, respectively, compared to *Lrrk2*^*+*/*+*^ cells (Fig. 2f, g). All together, these findings indicate that LRRK2 modulates inflammation acting upstream of pro-inflammatory mRNA transcription.

### *Lrrk2*^*−*/*−*^ primary microglia cells exhibit attenuated inflammatory response after priming with α-syn fibrils

**Fig. 3:**
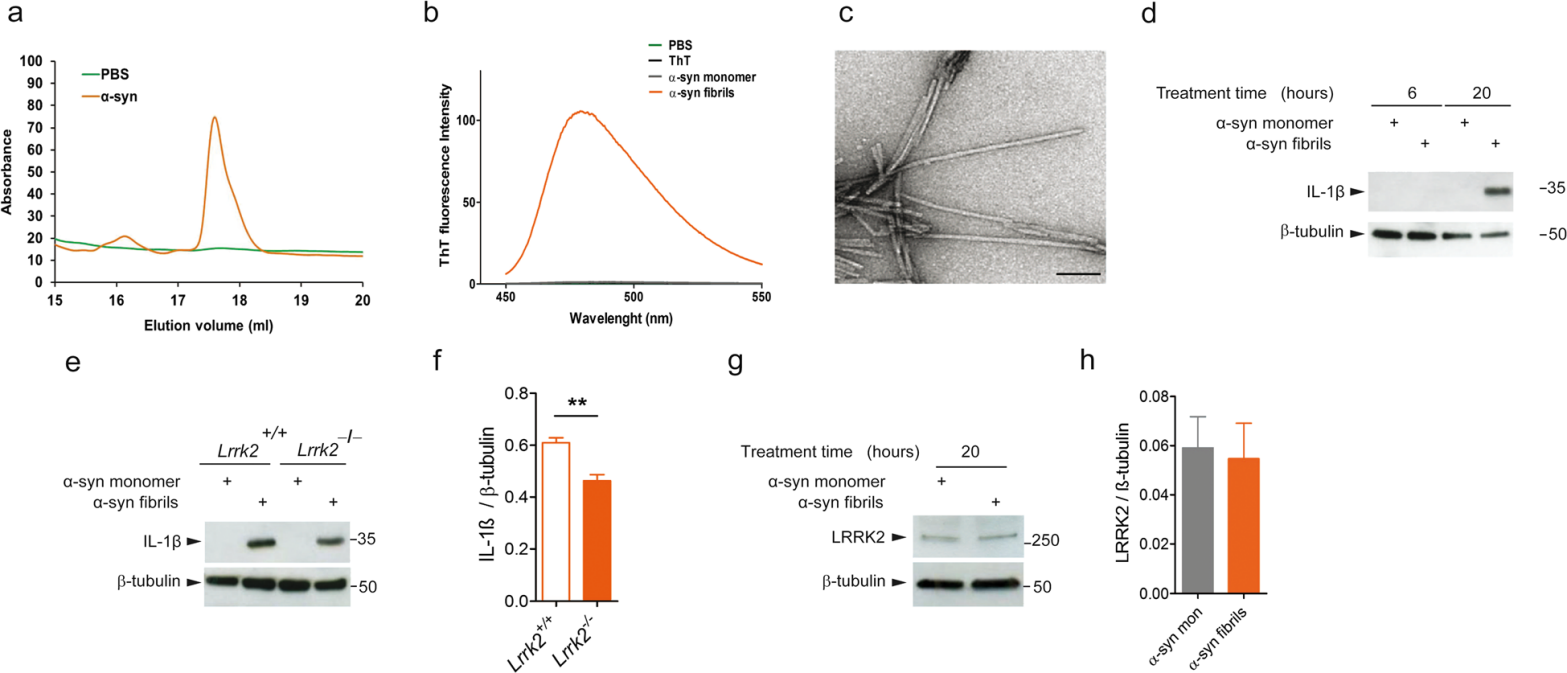
*Lrrk2* ^*−*/*−*^ primary microglia cells exhibit attenuated inflammation after α-syn fibrils priming. **a** After purification, α-syn was subjected to reverse phase HPLC chromatography. α-syn aggregation and fibrils formation were validated by Thioflavin assay (**b**) and TEM imaging (**c**). Scale bar 100 nm. **d** *Lrrk2* ^*+*/*+*^ microglia lysates treated with 25 μM α-syn fibrils (concentration before fibrillation) or 25 nM α-syn monomer as control for 6 and 20 h were subjected to immunoblotting using IL-1β antibody. **e** *Lrrk2* ^*+*/*+*^ *and Lrrk2* ^*−*/*−*^ microglia lysates treated with 25 μM α-syn fibrils (concentration before fibrillation) or 25 nM α-syn monomer as control were subjected to immunoblotting using IL-1β antibody. **f** Quantification of α-syn fibrils-mediated IL-1β expression is normalized for β-tubulin. Data are representative of four independent experiments (*bars* represent the mean ± SEM; unpaired *t* test; **p < 0.01). **g** *Lrrk2* ^*+*/*+*^ microglia lysates treated with 25 μM α-syn fibrils (concentration before fibrillation) or 25 nM α-syn monomer as control for 20 h were subjected to immunoblotting using LRRK2 antibody. **h** Quantification of LRRK2 expression is normalized for β-tubulin. Data are representative of three independent experiments (*bars* represent the mean ± SEM)

Multiple lines of evidence suggest that aggregated forms of α-syn released from dying neurons can activate microglia [39, 40]. Thus, we asked whether LRRK2 participates in the inflammatory response mediated by α-syn aggregated forms. To this end, we generated α-syn fibrils from recombinant α-syn produced using bacteria with reduced endotoxicity (Fig. 3a). α-Syn monomers were incubated for 15 days to induce aggregation, and fibrils formation was validated by both Thioflavin assay and TEM imaging (Fig 3b, c).

In analogy to the experiments with LPS, α-syn treatments were performed in 1 % FBS containing medium, a condition we observed to induce the maximal response in terms of IL-1β and COX-2 induction. Inflammation was triggered using 25 μM α-syn fibrils (expressed as initial concentration of monomer before fibrillation), and α-syn monomer at 25 nM was used as control. Considering that one fibril is estimated to contain ~10.000 monomers [41], 25 nM of monomeric α-syn corresponds to a ~10× molar excess of α-syn fibrils (2.5 nM). We treated *Lrrk2*^*+*/*+*^ microglia cells with 25 μM α-syn fibrils for 6 and 20 h and found that α-syn fibrils are able to induce the expression of pro-inflammatory IL-1β protein only after 20 h of treatment (Fig. 3d). Interestingly, when we examined IL-1β content in *Lrrk2*^*−*/*−*^ microglia cells, we observed a marked decreased of protein levels compared to *Lrrk2*^*+*/*+*^ microglia cells (Fig. 3e, f), indicating that LRRK2 is part of the molecular pathway trigged by α-syn fibrils, which culminates in microglia activation. Of note, LRRK2 steady state levels did not change upon treatment with α-syn fibrils for 20 h (Fig. 3g, h).

### LRRK2 does not influence p65 phosphorylation and p50 maturation upon LPS priming

**Fig. 4:**
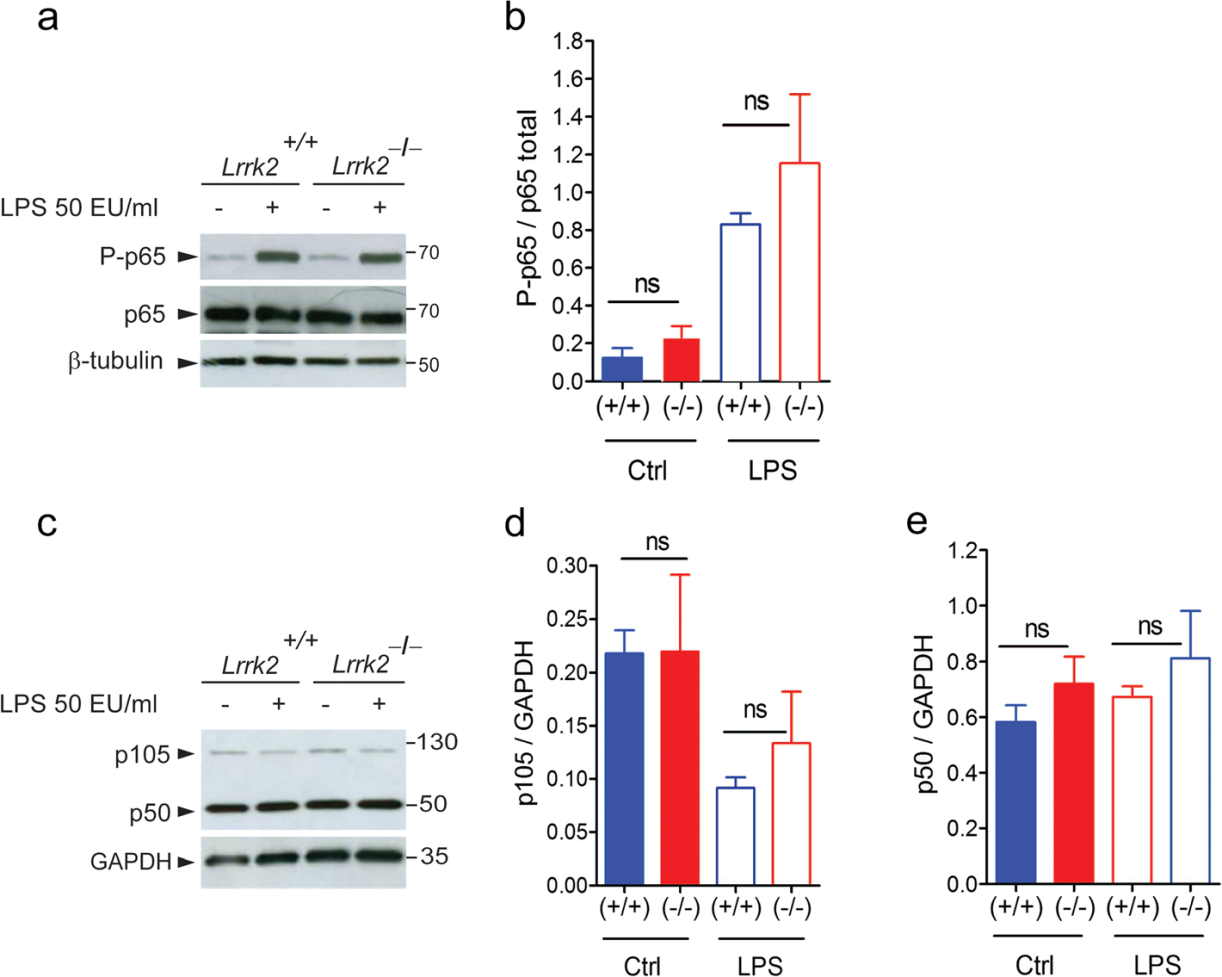
LRRK2 does not influence p65 phosphorylation and p50 maturation upon LPS priming. **a** *Lrrk2* ^+/+^ and *Lrrk2* ^*−*/*−*^ microglia lysates treated with LPS or PBS as control were subjected to immunoblotting using NF-κB P-p65 and p65 antibodies. **b** Quantification of P-p65 is normalized for p65 total protein. Data are representative of three independent experiments (*bars* represent the mean ± SEM; one-way ANOVA comparing all groups, Tukey’s multiple comparison post-hoc test). **c** *Lrrk2* ^+/+^ and *Lrrk2* ^*−*/*−*^ microglia lysates treated with LPS or PBS as control were subjected to immunoblotting using NF-κB p105/p50 antibody. Quantification of p105 (**d**) and p50 (**e**) proteins are normalized for GAPDH. Data are representative of three independent experiments, respectively, (*bars* represent the mean ± SEM; one-way ANOVA comparing all groups, Tukey’s multiple comparison post hoc test)

Having established that LRRK2 is a positive mediator of microglial inflammation triggered by LPS and, more importantly, fibrillar α-syn, we next asked which molecular mechanism might govern this process. Starting from the observation that (1) LRRK2 has been reported to influence NF-κB transcription activity [24], (2) both LPS [42] and aggregated α-syn [43] activate NF-κB signaling to induce inflammation, and (3) *Lrrk2*^−/−^ microglia cells display decreased pro-inflammatory mediators upstream of mRNA transcription (Fig. 2), we investigated whether cultured *Lrrk2*^−/−^ microglia cells exhibit alterations of the NF-κB pathway after an inflammatory stimulus. First, we asked whether LRRK2 influences p65 signaling after LPS-mediated inflammation. Both *Lrrk2*^*+*/*+*^ and *Lrrk2*^*−*/*−*^ microglia cells primed with LPS for 90 min showed a significant increase of p65 phosphorylation at S536, a readout of p65 activation [44], with no difference between the two genotypes (Fig. 4a, b), indicating that LRRK2 does not appear to influence p65 activation and the related signaling. Second, we analyzed the expression levels of both p50 and its p105 precursor in *Lrrk2*^*+*/*+*^ and *Lrrk2*^*−*/*−*^ primary microglia cells. Treatment with LPS (90 min) induced a reduction of p105 precursor and a trend of increment of the p50 mature form, as expected, with no difference between *Lrrk2*^*+*/*+*^ and *Lrrk2*^*−*/*−*^ primary microglia cells (Fig. 4c–e), suggesting that LRRK2 may not intervene in the maturation of p50.

### *Lrrk2*^*−*/*−*^ primary microglia cells exhibit increased levels of phosphorylated and nuclear NF-κB p50

**Fig. 5:**
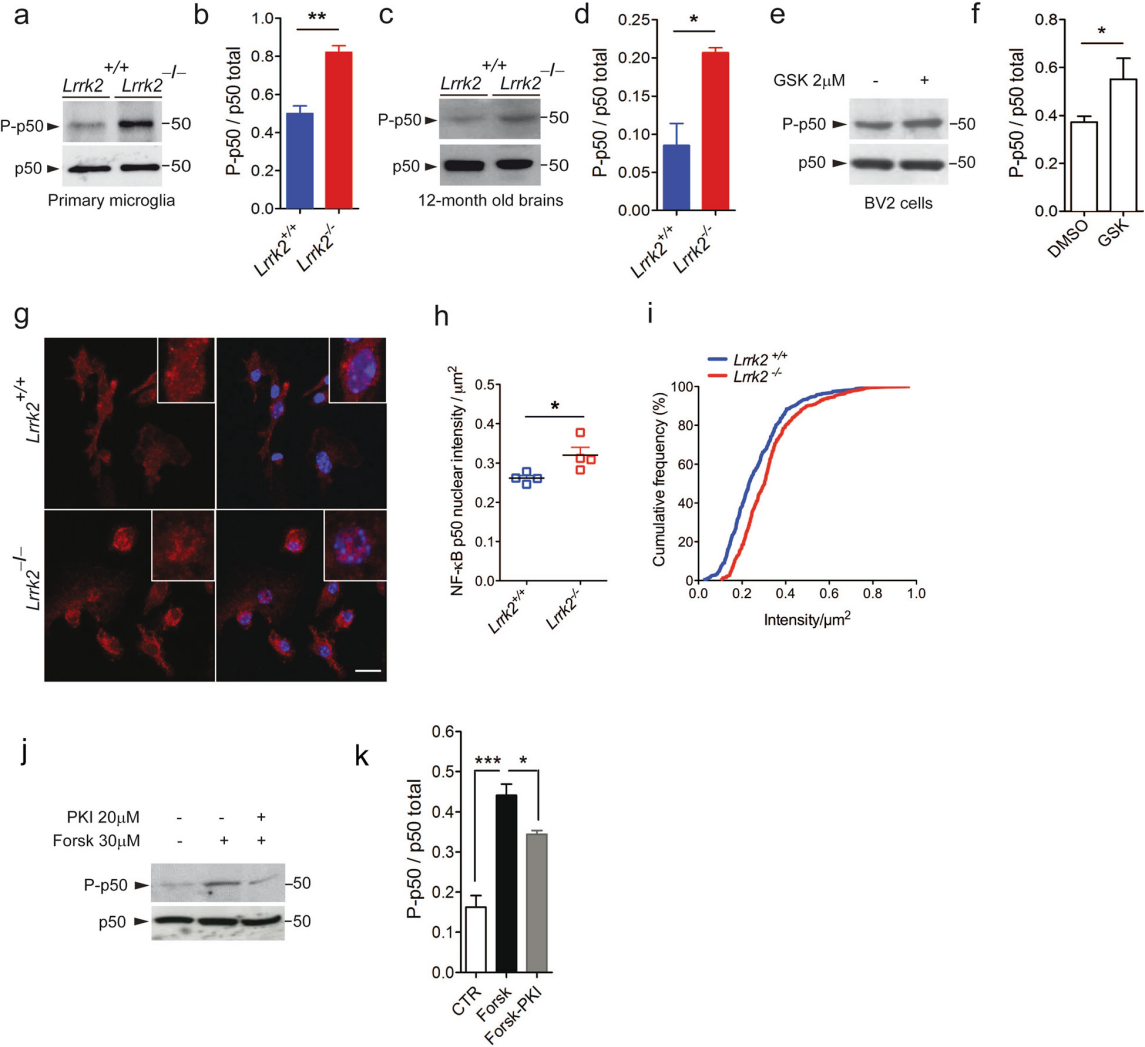
*Lrrk2* ^*−*/*−*^ primary microglia cells reported increased levels of phosphorylated and nuclear NF-κB p50 subunit under unstimulated conditions. **a** *Lrrk2* ^+/+^ and *Lrrk2* ^*−*/*−*^ microglia lysates were subjected to immunoblotting using NF-κB P-p50 and p50 total antibodies. **b** Quantification of P-p50 subunit is normalized for p50 total protein. Data are representative of three independent experiments (*bars* represent the mean ± SEM; unpaired *t* test; **p > 0.01). **c** *Lrrk2* ^+/+^ and *Lrrk2* ^*−*/*−*^ brain lysates were subjected to immunoblotting using NF-κB P-p50 and p50 total antibodies. **d** Quantification of P-p50 subunit is normalized for p50 total protein. Data are representative of three independent experiments (*bars* represent the mean ± SEM; unpaired *t* test; *p > 0.05). **e** BV2 cell lysates treated with GSK or DMSO as control were subjected to immunoblotting using NF-κB P-p50 and p50 total antibodies. **f** Quantification of P-p50 subunit is normalized for p50 total protein. Data are representative of three independent experiments (*bars* represent the mean ± SEM; unpaired *t* test; *p > 0.05). **g** Representative images of unstimulated *Lrrk2* ^+/+^ and *Lrrk2* ^*−*/*−*^ microglia cells stained for NF-κB p50 (*red*) and nuclei with Hoechst (*blue*). Scale bar 10 μm. **h** Quantification of nuclear NF-κB p50 shown as mean of fluorescence intensity from four independent experiments (~90 cells per experiment per genotype). Nuclear NF-κB p50 was calculated as nuclear fluorescence intensity divided by the nuclear area (μm^2^) (*bars* represent the mean ± SEM; unpaired *t* test; *p < 0.05). **i** Cumulative frequency distributions of nuclear p50 in *Lrrk2* ^+/+^ and *Lrrk2* ^*−/−*^ microglia cells (*n*_(*Lrrk2+/+*)_ = 352 and *n*_(*Lrrk2−/−*)_ = 377; Kolmogorov-Smirnov test, *p* = 0.004). **j** BV2 cell lysates treated with forskolin or PKI were subjected to immunoblotting using P-p50 and p50 total antibodies. **k** Quantification of P-p50 is normalized for p50 total protein. Data are representative of five independent experiments (*bars* represent the mean ± SEM; unpaired *t* test; *p > 0.05, ***p > 0.001)

Starting from the observation that DNA binding by p50:p50 homodimer is increased in BV2 cells when LRRK2 is knocked-down [24], we next asked whether LRRK2 impacts the NF-κB p50 inhibitory signaling. To this end, we analyzed the levels of p50 phosphorylation at S337 (P-p50) in *Lrrk2*^*+*/*+*^ and *Lrrk2*^*−*/*−*^ cells. Constitutive phosphorylation of S337 by PKA increases p50 affinity to DNA [45] maintaining stable negative regulation of NF-κB gene expression in the absence of extracellular stimulation [46]. Since LRRK2 was recently shown to negatively regulate PKA activation [47], we investigated whether S337 phosphorylation was altered in cultured *Lrrk2*^*−*/*−*^ microglia. As shown in (Fig. 5a, b), *Lrrk2*^*−*/*−*^ microglia cells exhibit ~65 % increase of P-p50 phosphorylation compared to *Lrrk2*^*+*/*+*^ microglia. In support of these findings, p50 phosphorylation is also enhanced in *Lrrk2*^*−*/*−*^ mouse brains (Fig. 5c, d) and in BV2 cells upon GSK pharmacological inhibition (Fig. 5e, f), suggesting that LRRK2 might modulate p50 affinity to DNA and the consequent repression of NF-κB-dependent gene transcription.

In contrast to p65:p50 heterodimer whose nuclear translocation is tightly regulated in presence of extracellular stimuli [48], p50:p50 homodimer is constitutively imported into the nucleus where it represses gene transcription [29]. Given that S337 phosphorylation controls p50:p50 binding to DNA [45], we then investigated whether the increased P-p50 observed in *Lrrk2*^*−*/*−*^ microglia correlates with an increased nuclear content of p50. To this aim, we quantified p50 nuclear fluorescence in unstimulated microglia cells. As shown in (Fig. 5g, h), *Lrrk2*^*−*/*−*^ microglia exhibit a higher proportion of nuclear p50 compared to *Lrrk2*^*+*/*+*^ cells. The cumulative frequency distribution analysis reveals that *Lrrk2*^*−*/*−*^ cells display p50 nuclear fluorescence consistently shifted toward higher intensity values with respect to *Lrrk2*^*+*/*+*^ cells (Fig. 5i), indicating a homogeneous increased of nuclear p50 in *Lrrk2*^*−*/*−*^ cells.

To verify that phosphorylation of p50 at S337 is a *bona fide* PKA phosphorylation site, we treated BV2 cells with forskolin, an activator of adenylate cyclase, and PKI, a specific PKA inhibitor [49]. Compared to untreated cells, forskolin induces ~twofold increment of p50 phosphorylation, which is attenuated by ~onefold in the presence of PKI (Fig. 5j, k).

**Fig. 6:**
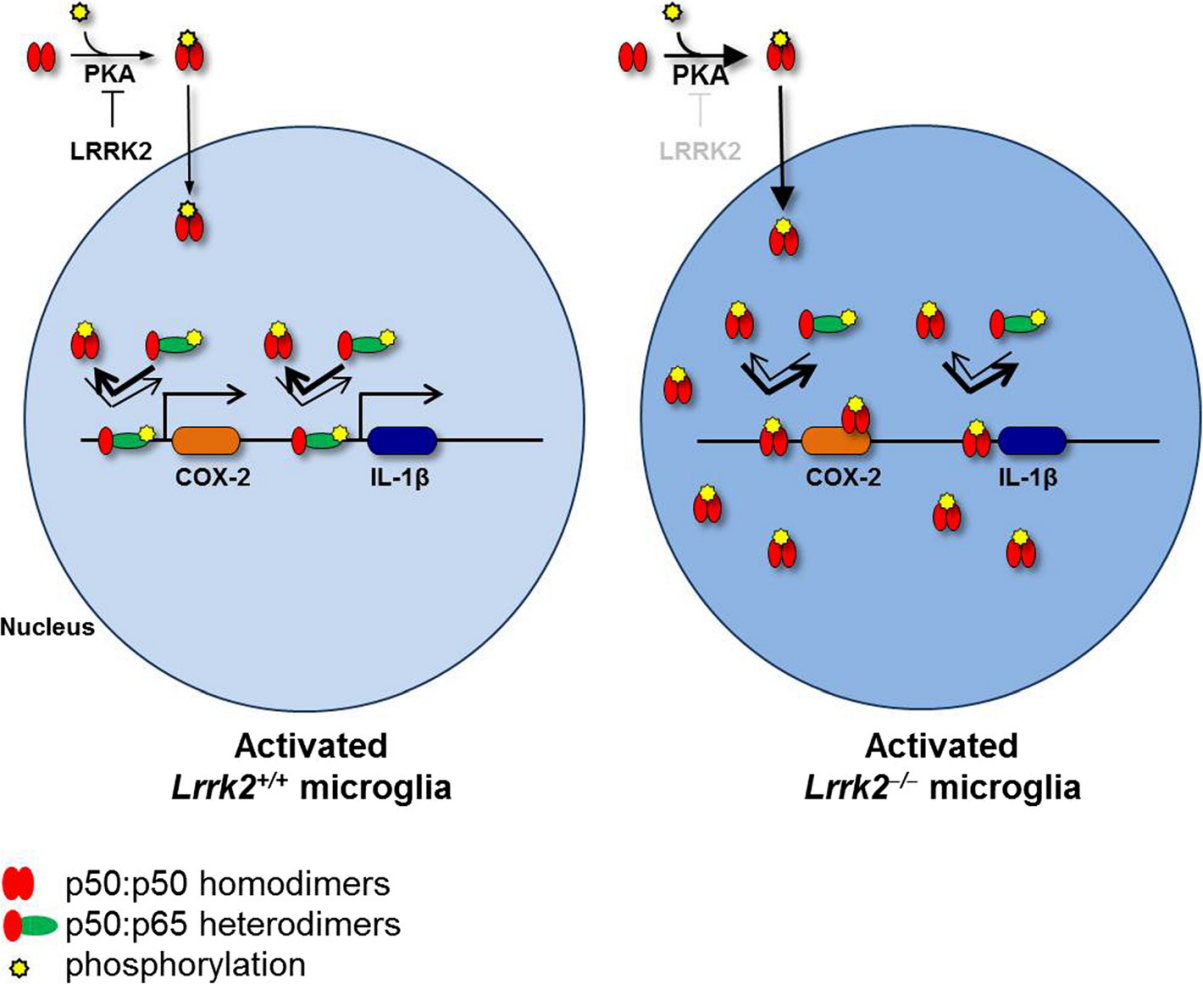
Schematic hypothesis. Genetic deletion of *Lrrk2* or inhibition of its kinase activity results in increased phosphorylation of NF-κB inhibitory subunit p50 at S337 with consequent accumulation of p50 in the nucleus. This abnormally higher proportion of nuclear P-p50 might hamper p65:p50 to efficiently bind to DNA and activate genes transcription upon LPS or α-syn-mediated inflammation

Taken together, our results suggest that LRRK2 modulates NF-κB p50 affinity to DNA through control of NF-κB p50 phosphorylation and consequent nuclear localization and suggest that the reduced inflammatory response of cells with LRRK2 pharmacological inhibition or deficiency may be due to an enhanced phosphorylation of NF-κB p50 inhibitory subunit bound to DNA (Fig. 6).

## Discussion

Over-activated microglia and chronic neuroinflammation may contribute to dopaminergic neuron degeneration and progression of PD [50]. While growing evidence supports a role of LRRK2 in activation of microglia cells [51, 52], its precise function in these cells remains poorly understood. In this study, using pharmacological inhibition in conjunction with *Lrrk2* knock-out cells, we provide evidence for a novel role of LRRK2 in the microglia.

LRRK2 kinase inhibition with IN-1 or Sunitinib has been previously reported to reduce transcription of inflammatory mRNAs induced by pathological conditions [33, 34, 53]. However, these inhibitors have shown significant off-target effects [35, 36], calling for independent evaluations of LRRK2 immunological function in the microglia. Here, we investigated the role of LRRK2 after LPS-mediated inflammation using the selective GSK2578215A inhibitor [38] and cultured *Lrrk2*^*−*/*−*^ primary microglia cells. We demonstrated that both BV2 treated with GSK and *Lrrk2*^*−*/*−*^microglia cells exhibit significant attenuation of inflammation at both transcription and translation levels, supporting the notion that the kinase activity of LRRK2 acts upstream of inflammatory mRNA transcription after LPS priming.

Neuroinflammation is a well-described condition in parkinsonian brains [54]. Dopaminergic neurons of the substantia nigra pars compacta, which are preferentially depleted during disease, are surrounded by an abnormally high concentration of microglia cells compared to other brain regions [55], making these neurons more susceptible to an inflammatory insult. Among the endogenous agents that might be competent of activating microglia cells, α-syn is a top candidate. It has been recently shown that α-syn induces microglia activation by engaging the heterodimer TLR1/2 (Toll-like receptors 1/2), which triggers nuclear translocation and activation of NF-κB [43]. Additional studies in vitro [40, 56] and in vivo [57] reported an increased expression of TLR2 and 4, supporting the notion that α-syn aggregated forms switch on microglia through TLRs. Thus, one possibility is that α-syn released from dying dopaminergic neurons locally activates the abundant microglia through the TLR/NF-κB pathway. This causes the release of pro-inflammatory cytokines, which in turn may signal back to neurons, initiating a vicious circle. In this scenario, LRRK2, which is a positive modulator of neuroinflammation, could mediate and contribute to α-syn-dependent microglia activation and neuroinflammation. To test this hypothesis, we asked whether LRRK2 is able to modulate the inflammatory response mediated by α-syn. Our data demonstrate that *Lrrk2*^*−*/*−*^ microglia exhibit reduced levels of IL-1β cytokine when exposed to α-syn fibrils compared to *Lrrk2*^*+*/*+*^ cells. In line with our findings, *Lrrk2*^*−*/*−*^ rats exhibit a reduced fraction of activated microglia and are more resistant to dopaminergic neurodegeneration after injection of rAAV2/1–α-syn in the substantia nigra compared to wild-type rats [58]. Of note, pathological or inhibited LRRK2 activity in microglia cells augments or reduces neuronal toxicity, respectively, supporting the notion that neurodegeneration might be triggered or amplified by LRRK2-dependent neuroinflammation [33, 59]. Thus, it is tempting to speculate that LRRK2 activity contributes to sustainment of neuroinflammation and that pharmacological inhibition treatments may be effective at attenuating chronic neuroinflammation and neurodegeneration in PD patients. In this context, several groups intensely worked to identify LRRK2 inhibitors with good physiochemical and pharmacokinetic properties, selectivity and blood-brain barrier permeability, against both kinase [60–63] and GTPase activities [64]. While LRRK2 inhibition has been proven to be effective at reducing pathological LRRK2 phenotypes [65], other studies showed that *Lrrk2* knock-out mice or non-human primates treated with chronic doses of LRRK2 inhibitors exhibit side effects and morphologic changes in peripheral tissue [66, 67]. Thus, additional investigations are necessary to determine the appropriate pharmacological doses to switch off the enhanced pathological kinase function preserving LRRK2 physiological activity.

Of note, few studies showed that stimulation of immune cells induces LRRK2 expression [34, 59, 68]. Here, we found that cultured microglia cells treated with LPS for 5 h or α-syn fibrils for 20 h did not exhibit a significant increase in LRRK2 protein expression, possibly indicating that LRRK2 behaves differently under distinct stimulation conditions and/or immune cell types.

We also provided mechanistic insights into LRRK2 modulation of microglial inflammation. Given that NF-κB transcription activity has been reported to be affected in BV2 cells with LRRK2 knock-down after LPS priming [24] and that both LPS [42] and aggregated α-syn forms [43] activate NF-κB signaling to induce inflammation, we investigated whether *Lrrk2*^−/−^ and inhibited microglia cells exhibit alterations in the NF-κB pathway. We focused on the canonical NF-κB pathway, the p65:p50 signaling, and found that both p50 phosphorylated at S337 and its nuclear localization are enhanced in cultured *Lrrk2*^*−*/*−*^microglia under unstimulated conditions. We demonstrated this LRRK2-mediated effect on P-p50 also in BV2 cells with LRRK2 kinase inhibition and *ex vivo* using 12-month old *Lrrk2*^*−*/*−*^ mouse brains. It has been previously reported that p50 phosphorylation at S337 by PKA possesses higher affinity to DNA [45] and represses NF-κB target gene in absence of extracellular stimuli [46]. Following stimulation, p50:p65 heterodimers translocate into the nucleus, compete, and displace DNA-bound p50:p50 to initiate mRNAs transcription [27]. Our results suggest that the reduced inflammatory response of cells with LRRK2 pharmacological inhibition or deficiency may be mediated by the excessive phosphorylation of NF-κB inhibitory p50 subunit. We propose that an abnormally higher proportion of P-p50 in the nucleus of *Lrrk2*^−*/*−^ or LRRK2-inhibited cells hampers p65:p50 binding to DNA and activation of gene transcription (Fig. 6). Coherently, with the hypothesis proposed here, Kim and colleagues reported that immortalized BV2 microglia with LRRK2 knock-down display more p50:p50 bound to DNA compared to wild-type cells in control condition and also after LPS treatment [24]. Taken the results previously reported and our findings together, LRRK2 kinase activity may modulate the induction of pro-inflammatory mediators by a negative regulation of PKA activity. Supporting a functional interaction between these two proteins, LRRK2 has been recently reported to act as a negative regulator of PKA signaling [47]. Specifically, the authors proposed LRRK2 as a novel AKAP, demonstrating that a lack of LRRK2 promoted synaptic translocation of PKA and increased PKA-mediated phosphorylation of cofilin and glutamate receptor GluR1, with consequent abnormal synaptogenesis [47].

## Conclusions

Overall, our study validates LRRK2 kinase activity as a positive modulator of inflammation in microglia cells. LRRK2 activity negatively regulates NF-κB p50 phosphorylation and nuclear translocation under physiological conditions; this might be one molecular mechanism underlying the reduction of NF-κB target-genes transcription during inflammatory stimuli. This study expands our understanding of LRRK2 function in microglia cells and suggests that additional research should be directed at identifying LRRK2 inhibition doses able to reduce LRRK2 pathological activity preserving its physiological function. Lowering LRRK2 kinase activity may allow to attenuate microglia activation and chronic neuroinflammation in PD patients.

## Abbreviations

COR: C-terminus of ROC
COX-2: cyclooxygenase-2
EU: endotoxin units
FBS: fetal bovine serum
Forsk: Forskolin
GSK: GSK2578215A
IL-1β: interleukin-1β
LPS: lipopolysaccharide
LRRK2: leucine-rich repeat kinase 2
NF-κB: nuclear factor kappa-B
PBS: phosphate-buffered saline
PD: Parkinson’s disease
ROC: Ras of complex proteins
ROCO: Ras of complex
RT: room temperature
TEM: transmission electron microscopy
α-syn: α-synuclein

## References

[1] Paisan-Ruiz C, Jain S, Evans EW, Gilks WP, Simon J, van der Brug M (2004). Cloning of the gene containing mutations that cause PARK8-linked Parkinson’s disease. Neuron.

[2] Zimprich A, Biskup S, Leitner P, Lichtner P, Farrer M, Lincoln S (2004). Mutations in LRRK2 cause autosomal-dominant parkinsonism with pleomorphic pathology. Neuron.

[3] Marin I (2006). The Parkinson disease gene LRRK2: evolutionary and structural insights. Mol Biol Evol.

[4] Goldwurm S, Di Fonzo A, Simons EJ, Rohe CF, Zini M, Canesi M (2005). The G6055A (G2019S) mutation in LRRK2 is frequent in both early and late onset Parkinson’s disease and originates from a common ancestor. J Med Genet.

[5] Greggio E, Jain S, Kingsbury A, Bandopadhyay R, Lewis P, Kaganovich A (2006). Kinase activity is required for the toxic effects of mutant LRRK2/dardarin. Neurobiol Dis.

[6] West AB, Moore DJ, Biskup S, Bugayenko A, Smith WW, Ross CA (2005). Parkinson’s disease-associated mutations in leucine-rich repeat kinase 2 augment kinase activity. Proc Natl Acad Sci U S A.

[7] Sheng Z, Zhang S, Bustos D, Kleinheinz T, Le Pichon CE, Dominguez SL (2012). Ser1292 autophosphorylation is an indicator of LRRK2 kinase activity and contributes to the cellular effects of PD mutations. Sci Transl Med.

[8] Smith WW, Pei Z, Jiang H, Dawson VL, Dawson TM, Ross CA (2006). Kinase activity of mutant LRRK2 mediates neuronal toxicity. Nat Neurosci.

[9] Cirnaru MD, Marte A, Belluzzi E, Russo I, Gabrielli M, Longo F (2014). LRRK2 kinase activity regulates synaptic vesicle trafficking and neurotransmitter release through modulation of LRRK2 macro-molecular complex. Front Mol Neurosci.

[10] Matta S, Van Kolen K, da Cunha R, van den Bogaart G, Mandemakers W, Miskiewicz K (2012). LRRK2 controls an EndoA phosphorylation cycle in synaptic endocytosis. Neuron.

[11] Kett LR, Boassa D, Ho CC, Rideout HJ, Hu J, Terada M (2012). LRRK2 Parkinson disease mutations enhance its microtubule association. Hum Mol Genet.

[12] Caesar M, Zach S, Carlson CB, Brockmann K, Gasser T, Gillardon F (2013). Leucine-rich repeat kinase 2 functionally interacts with microtubules and kinase-dependently modulates cell migration. Neurobiol Dis.

[13] Civiero L, Cirnaru MD, Beilina A, Rodella U, Russo I, Belluzzi E, et al. LRRK2 interacts with PAK6 to control neurite complexity in mammalian brain. J Neurochem. 2015. 10.1111/jnc.13369 PMC4715492 26375402

[14] Papkovskaia TD, Chau KY, Inesta-Vaquera F, Papkovsky DB, Healy DG, Nishio K (2012). G2019S leucine-rich repeat kinase 2 causes uncoupling protein-mediated mitochondrial depolarization. Hum Mol Genet.

[15] Wang X, Yan MH, Fujioka H, Liu J, Wilson-Delfosse A, Chen SG (2012). LRRK2 regulates mitochondrial dynamics and function through direct interaction with DLP1. Hum Mol Genet.

[16] Ho CC, Rideout HJ, Ribe E, Troy CM, Dauer WT (2009). The Parkinson disease protein leucine-rich repeat kinase 2 transduces death signals via Fas-associated protein with death domain and caspase-8 in a cellular model of neurodegeneration. J Neurosci.

[17] Orenstein SJ, Kuo SH, Tasset I, Arias E, Koga H, Fernandez-Carasa I (2013). Interplay of LRRK2 with chaperone-mediated autophagy. Nat Neurosci.

[18] Gomez-Suaga P, Luzon-Toro B, Churamani D, Zhang L, Bloor-Young D, Patel S (2012). Leucine-rich repeat kinase 2 regulates autophagy through a calcium-dependent pathway involving NAADP. Hum Mol Genet.

[19] Gardet A, Benita Y, Li C, Sands BE, Ballester I, Stevens C (2010). LRRK2 is involved in the IFN-gamma response and host response to pathogens. J Immunol.

[20] Schapansky J, Nardozzi JD, Felizia F, LaVoie MJ (2014). Membrane recruitment of endogenous LRRK2 precedes its potent regulation of autophagy. Hum Mol Genet.

[21] Barrett JC, Hansoul S, Nicolae DL, Cho JH, Duerr RH, Rioux JD (2008). Genome-wide association defines more than 30 distinct susceptibility loci for Crohn’s disease. Nat Genet.

[22] Zhang FR, Huang W, Chen SM, Sun LD, Liu H, Li Y (2009). Genomewide association study of leprosy. N Engl J Med.

[23] Liu Z, Lee J, Krummey S, Lu W, Cai H, Lenardo MJ (2011). The kinase LRRK2 is a regulator of the transcription factor NFAT that modulates the severity of inflammatory bowel disease. Nat Immunol.

[24] Kim B, Yang MS, Choi D, Kim JH, Kim HS, Seol W (2012). Impaired inflammatory responses in murine Lrrk2-knockdown brain microglia. PLoS One.

[25] Tak PP, Firestein GS (2001). NF-kappaB: a key role in inflammatory diseases. J Clin Invest.

[26] Ghosh S, May MJ, Kopp EB (1998). NF-kappa B and Rel proteins: evolutionarily conserved mediators of immune responses. Annu Rev Immunol.

[27] Zhong H, May MJ, Jimi E, Ghosh S (2002). The phosphorylation status of nuclear NF-kappa B determines its association with CBP/p300 or HDAC-1. Mol Cell.

[28] Cheng CS, Feldman KE, Lee J, Verma S, Huang DB, Huynh K (2011). The specificity of innate immune responses is enforced by repression of interferon response elements by NF-kappaB p50. Sci Signal.

[29] Ten RM, Paya CV, Israel N, Le Bail O, Mattei MG, Virelizier JL (1992). The characterization of the promoter of the gene encoding the p50 subunit of NF-kappa B indicates that it participates in its own regulation. EMBO J.

[30] Napetschnig J, Wu H (2013). Molecular basis of NF-kappaB signaling. Annu Rev Biophys.

[31] Cognet I, de Coignac AB, Magistrelli G, Jeannin P, Aubry JP, Maisnier-Patin K (2003). Expression of recombinant proteins in a lipid A mutant of *Escherichia coli* BL21 with a strongly reduced capacity to induce dendritic cell activation and maturation. J Immunol Methods.

[32] Codolo G, Plotegher N, Pozzobon T, Brucale M, Tessari I, Bubacco L (2013). Triggering of inflammasome by aggregated alpha-synuclein, an inflammatory response in synucleinopathies. PLoS One.

[33] Marker DF, Puccini JM, Mockus TE, Barbieri J, Lu SM, Gelbard HA (2012). LRRK2 kinase inhibition prevents pathological microglial phagocytosis in response to HIV-1 Tat protein. J Neuroinflammation.

[34] Moehle MS, Webber PJ, Tse T, Sukar N, Standaert DG, DeSilva TM (2012). LRRK2 inhibition attenuates microglial inflammatory responses. J Neurosci.

[35] Dzamko N, Deak M, Hentati F, Reith AD, Prescott AR, Alessi DR (2010). Inhibition of LRRK2 kinase activity leads to dephosphorylation of Ser(910)/Ser(935), disruption of 14-3-3 binding and altered cytoplasmic localization. Biochem J.

[36] Luerman GC, Nguyen C, Samaroo H, Loos P, Xi H, Hurtado-Lorenzo A (2014). Phosphoproteomic evaluation of pharmacological inhibition of leucine-rich repeat kinase 2 reveals significant off-target effects of LRRK-2-IN-1. J Neurochem.

[37] Deng X, Dzamko N, Prescott A, Davies P, Liu Q, Yang Q (2011). Characterization of a selective inhibitor of the Parkinson’s disease kinase LRRK2. Nat Chem Biol.

[38] Reith AD, Bamborough P, Jandu K, Andreotti D, Mensah L, Dossang P (2012). GSK2578215A; a potent and highly selective 2-arylmethyloxy-5-substitutent-N-arylbenzamide LRRK2 kinase inhibitor. Bioorg Med Chem Lett.

[39] Zhang W, Wang T, Pei Z, Miller DS, Wu X, Block ML (2005). Aggregated alpha-synuclein activates microglia: a process leading to disease progression in Parkinson’s disease. FASEB J.

[40] Kim C, Ho DH, Suk JE, You S, Michael S, Kang J (2013). Neuron-released oligomeric alpha-synuclein is an endogenous agonist of TLR2 for paracrine activation of microglia. Nat Commun.

[41] van Gestel J, de Leeuw SW (2006). A statistical-mechanical theory of fibril formation in dilute protein solutions. Biophys J.

[42] Lu YC, Yeh WC, Ohashi PS (2008). LPS/TLR4 signal transduction pathway. Cytokine.

[43] Daniele SG, Beraud D, Davenport C, Cheng K, Yin H, Maguire-Zeiss KA (2015). Activation of MyD88-dependent TLR1/2 signaling by misfolded alpha-synuclein, a protein linked to neurodegenerative disorders. Sci Signal.

[44] Wan F, Lenardo MJ (2009). Specification of DNA binding activity of NF-kappaB proteins. Cold Spring Harb Perspect Biol.

[45] Guan H, Hou S, Ricciardi RP (2005). DNA binding of repressor nuclear factor-kappaB p50/p50 depends on phosphorylation of Ser337 by the protein kinase A catalytic subunit. J Biol Chem.

[46] Hou S, Guan H, Ricciardi RP (2003). Phosphorylation of serine 337 of NF-kappaB p50 is critical for DNA binding. J Biol Chem.

[47] Parisiadou L, Yu J, Sgobio C, Xie C, Liu G, Sun L (2014). LRRK2 regulates synaptogenesis and dopamine receptor activation through modulation of PKA activity. Nat Neurosci.

[48] Baeuerle PA, Baltimore D (1996). NF-kappa B: ten years after. Cell.

[49] Glass DB, Lundquist LJ, Katz BM, Walsh DA (1989). Protein kinase inhibitor-(6–22)-amide peptide analogs with standard and nonstandard amino acid substitutions for phenylalanine 10. Inhibition of cAMP-dependent protein kinase. J Biol Chem.

[50] Hirsch EC, Hunot S (2009). Neuroinflammation in Parkinson’s disease: a target for neuroprotection?. Lancet Neurol.

[51] Russo I, Bubacco L, Greggio E (2014). LRRK2 and neuroinflammation: partners in crime in Parkinson’s disease?. J Neuroinflammation.

[52] Schapansky J, Nardozzi JD, LaVoie MJ (2015). The complex relationships between microglia, alpha-synuclein, and LRRK2 in Parkinson’s disease. Neuroscience.

[53] Puccini JM, Marker DF, Fitzgerald T, Barbieri J, Kim CS, Miller-Rhodes P (2015). Leucine-rich repeat kinase 2 modulates neuroinflammation and neurotoxicity in models of human immunodeficiency virus 1-associated neurocognitive disorders. J Neurosci.

[54] Tansey MG, McCoy MK, Frank-Cannon TC (2007). Neuroinflammatory mechanisms in Parkinson’s disease: potential environmental triggers, pathways, and targets for early therapeutic intervention. Exp Neurol.

[55] Kim WG, Mohney RP, Wilson B, Jeohn GH, Liu B, Hong JS (2000). Regional difference in susceptibility to lipopolysaccharide-induced neurotoxicity in the rat brain: role of microglia. J Neurosci.

[56] Fellner L, Irschick R, Schanda K, Reindl M, Klimaschewski L, Poewe W (2013). Toll-like receptor 4 is required for alpha-synuclein dependent activation of microglia and astroglia. Glia.

[57] Watson MB, Richter F, Lee SK, Gabby L, Wu J, Masliah E (2012). Regionally-specific microglial activation in young mice over-expressing human wildtype alpha-synuclein. Exp Neurol.

[58] Daher JP, Volpicelli-Daley LA, Blackburn JP, Moehle MS, West AB (2014). Abrogation of alpha-synuclein-mediated dopaminergic neurodegeneration in LRRK2-deficient rats. Proc Natl Acad Sci U S A.

[59] Gillardon F, Schmid R, Draheim H (2012). Parkinson’s disease-linked leucine-rich repeat kinase 2(R1441G) mutation increases proinflammatory cytokine release from activated primary microglial cells and resultant neurotoxicity. Neuroscience.

[60] Hatcher JM, Zhang J, Choi HG, Ito G, Alessi DR, Gray NS (2015). Discovery of a pyrrolopyrimidine (JH-II-127), a highly potent, selective, and brain penetrant LRRK2 inhibitor. ACS Med Chem Lett.

[61] Koshibu K, van Asperen J, Gerets H, Garcia-Ladona J, Lorthioir O, Courade JP (2015). Alternative to LRRK2-IN-1 for pharmacological studies of Parkinson’s disease. Pharmacology.

[62] Henderson JL, Kormos BL, Hayward MM, Coffman KJ, Jasti J, Kurumbail RG (2015). Discovery and preclinical profiling of 3-[4-(morpholin-4-yl)-7H-pyrrolo[2,3-d]pyrimidin-5-yl]benzonitrile (PF-06447475), a highly potent, selective, brain penetrant, and in vivo active LRRK2 kinase inhibitor. J Med Chem.

[63] Munoz L, Kavanagh ME, Phoa AF, Heng B, Dzamko N, Chen EJ (2015). Optimisation of LRRK2 inhibitors and assessment of functional efficacy in cell-based models of neuroinflammation. Eur J Med Chem.

[64] Li T, He X, Thomas JM, Yang D, Zhong S, Xue F (2015). A novel GTP-binding inhibitor, FX2149, attenuates LRRK2 toxicity in Parkinson’s disease models. PLoS One.

[65] Daher JP, Abdelmotilib HA, Hu X, Volpicelli-Daley LA, Moehle MS, Fraser KB (2015). Leucine-rich repeat kinase 2 (LRRK2) pharmacological inhibition abates alpha-synuclein gene-induced neurodegeneration. J Biol Chem.

[66] Fuji RN, Flagella M, Baca M, MA SB, Brodbeck J, Chan BK (2015). Effect of selective LRRK2 kinase inhibition on nonhuman primate lung. Sci Transl Med.

[67] Miklavc P, Ehinger K, Thompson KE, Hobi N, Shimshek DR, Frick M (2014). Surfactant secretion in LRRK2 knock-out rats: changes in lamellar body morphology and rate of exocytosis. PLoS One.

[68] Hakimi M, Selvanantham T, Swinton E, Padmore RF, Tong Y, Kabbach G (2011). Parkinson’s disease-linked LRRK2 is expressed in circulating and tissue immune cells and upregulated following recognition of microbial structures. J Neural Transm (Vienna).

