# Erratum to: Leucine-rich repeat kinase 2 positively regulates inflammation and down-regulates NF-κB p50 signaling in cultured microglia cells

**DOI:** 10.1186/s12974-016-0535-5

**Published:** 2016-04-01

**Authors:** Isabella Russo, Giulia Berti, Nicoletta Plotegher, Greta Bernardo, Roberta Filograna, Luigi Bubacco, Elisa Greggio

**Affiliations:** Department of Biology, University of Padova, Padova, Italy; Present address: Department of Laboratory Medicine, Karolinska Institute, Stockholm, Sweden

## Erratum

After publication of this work [1], it was noted that a funding agency was missed within the acknowledgements section. The correct version of the acknowledgements section can be found below:
